# Risk Factors for COVID-19 Infection among Healthcare Workers in North-East Nigeria

**DOI:** 10.3390/healthcare10101919

**Published:** 2022-09-30

**Authors:** Roland I. Stephen, Jamiu Olumoh, Jennifer Tyndall, Oyelola Adegboye

**Affiliations:** 1 Department of Internal Medicine, Modibbo Adama University Teaching Hospital, Yola 640001, Adamawa State, Nigeria; 2 Department of Mathematics and Statistics, American University of Nigeria, Yola 640001, Adamawa State, Nigeria; 3 Department of Natural and Environmental Sciences, American University of Nigeria, Yola 640001, Adamawa State, Nigeria; 4 Public Health and Tropical Medicine, College of Public Health, Medical and Veterinary Sciences, James Cook University, Townsville, QLD 4811, Australia; 5 World Health Organization Collaborating Center for Vector-Borne and Neglected Tropical Diseases, College of Public Health, Medical and Veterinary Sciences, James Cook University, Townsville, QLD 4811, Australia; 6 Australian Institute of Tropical Health and Medicine, James Cook University, Townsville, QLD 4811, Australia

**Keywords:** COVID-19, healthcare workers, clinical staff, personal protective equipment

## Abstract

Healthcare workers (HCWs) face an unprecedented higher risk of COVID-19 infection due to their work and exposure. In this study, we aim to examine the associated risk factors for COVID-19 infection among HCWs in North-East Nigeria. We used data collected retrospectively among a cohort of clinical and non-clinical HCWs in six healthcare facilities in Adamawa State, Nigeria. We estimated the marginal probability of COVID-19 infection among HWCs using alternating logistic regression via the generalized estimating equations (GEE) approach. Among the 318 HCWs, 178 (55.97%) were males, mean (±SD) age was 36.81 (±8.98), 237 (74.76%) were clinical, and 80 (25.24) were non-clinical staff. The overall prevalence of COVID-19 was 16.67% among HCWs. After adjusting for other variables in the model, our results showed that clinical staff had a 5-fold higher risk of COVID-19 infection than non-clinical staff (aOR = 5.07, 95% CI: 1.32–19.52). Moreover, significant exposure risk factors for COVID-19 infection for HCWs increase with age, time spent attending to patients, caring for COVID-19 patients, and having worked with COVID-19 samples, while the risk decreases with the use of an N95 mask. Our findings suggested that the burden of COVID-19 infection is higher for clinical staff than non-clinical staff, and increasing age contributed to the increased risk.

## 1. Introduction

The outbreak of the COVID-19 pandemic in December 2019 marked yet another landmark in the history of respiratory epidemics. Globally, the burden of COVID-19 constitutes a public health crisis on the healthcare infrastructure, including healthcare workers (HCWs). The first instance of COVID-19 in Nigeria was documented on 27th February 2020 [1,2]; as of the 11th of July 2022, more than 552 million COVID-19 cases and 6.3 million deaths (case fatality rate of 1.15%) have been reported globally (https://covid19.who.int/, accessed on 11 July 2022). The shock waves of the pandemic have impacted the world economy tremendously [3,4].

HCWs are on the frontline as an indispensable workforce in effectively managing and containing the pandemic. However, due to the mechanism of COVID-19 transmission (the virus leverages its spread through respiratory droplets, interpersonal contacts, contaminated surfaces, and fomites), these come with a higher occupational health risk [5]. The occupational transmission of the disease is taking its toll on the HCWs, which constitutes a critical integral component in the whole transmission dynamics of the pandemic. Previous studies have shown that the prevalence of COVID-19 is relatively higher among HCWs than in the general population largely due to the peculiarity of the HCWs risk exposure and vulnerability [6,7]. A prospective cohort study among HCWS in the US and UK found that inadequate access and reusing of personal protective equipment (PPE) increased the risk of COVID-19 infection [8]. A study in southern Nigeria found the prevalence of COVID-19 among HCWs to be 15.2% while highlighting that a relatively increased risk of disease rests on the clinical HCWs who deal directly with patients in Hospital settings [9]. Overcrowding of health facilities, inadequate PPEs, work overload, poor infection and prevention practices and inadequate isolation room facilities were risk factors for COVID-19 infection in hospital settings [10]. In their study, Ogboghodo et al. [11] established an epidemiological linkage between COVID-19 cases among HCWs to other COVID-19 positive fellow HCWs, community members, and in-hospital patients in a southern Nigerian-based study. The study further asserts that the clinical HCWs face double risks of infection in the hospital or the community. COVID-19 infected HCWs constitute a hazard to the hospital community and society because of their potential to be a super spreader. Most published studies dealing with the occupational risk of COVID-19 among HCWs were conducted in well-resourced climes and temperate regions. Evaluating these risks in HCWs working in poor resources, low-income climes, and the Sahel region is critical, hence the import of this study. The objectives of this study are two-fold; (1) to examine the risk factors associated with COVID-19 infections among HCWs based on their level of exposure, duration and contacts, and (2) To ascertain the impact of inadequate PPE and COVID-19 infection among HCWs.

## 2. Materials and Methods

### 2.1. Study Area, Design and Data Collection

**Figure 1:**
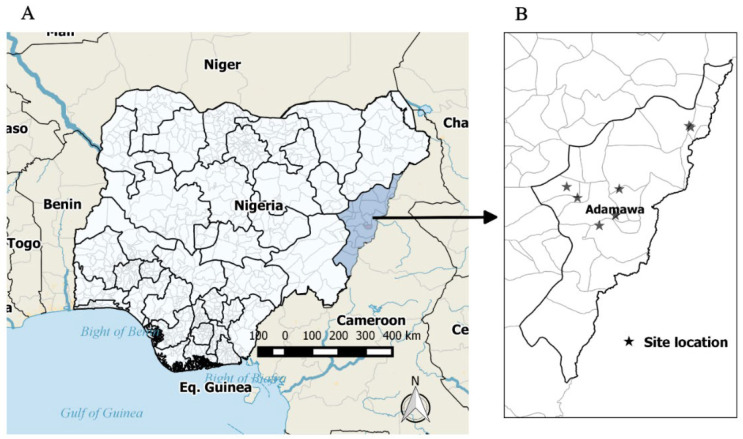
(**A**) Map of Nigeria showing the study sites in Adamawa state; (**B**) Study sites.

This study was based on retrospective cross-sectional data collected across selected sites in Adamawa state, North-East Nigeria. The sites were chosen to reflect the state’s rural and urban areas in five local government areas: Yola South, Yola North, Numan, Mubi South, and Mubi North (Figure 1). A survey was carried out from April 2020 to January 2021 in four public and three private health facilities within the study area. This period coincides with the first wave (week 9–43, 2020) and second (week 44, 2020 to week 13, 2021) wave of COVID-19 in Nigeria. The four largest public hospitals comprise two COVID-19 treatment centers. See the Supplementary Materials for the questionnaire. Eligible HCWs (staff of healthcare facilities) were classified as “clinical staff” if they are involved in direct patient care as part of their regular routine, and non-clinical staff are those not involved in patient care and with no opportunity for patient contact during their regular work routine [12].

Trained study staff and data collectors administered questionnaires in all locations. The survey questions include demographic characteristics, exposures, and COVID-19 infection test results. Trained laboratory scientists fully kited in their PPEs insert the swap stick into the nasopharynx of HCWs who present with symptoms suspicious of COVID-19 or had significant exposure. The swap was then subjected to polymerase Chain reaction (PCR) testing. Turnaround time was 48–72 h.

Based on COVID-19 infection prevalence rate (*p*) of 5—29% among HCWs [13,14,15], 5% precision and a 95% confidence level, we estimated a sample of 73 to 317 HCWs is adequate for this study using the formula [16] below:

$$n=\frac{Z^{2}p\left(1-p\right)}{d^{2}}$$

### 2.2. Data Analysis

Categorical variables were presented in frequencies and percentages, and continuous variables in mean and standard deviations. Chi-square and *t*-test tests were used to compare participant baseline characteristics as appropriate. To identify the associated risk factors for an HCW testing positive for COVID-19 or not, we fitted a marginal model via alternating logistic regression (ALR), an alternative to generalized estimating equations (GEE) was used. Given the clustered nature of the data, ALR allows the modeling of association between pairs of responses using a within-cluster (hospital) pair-wise odds ratio (POR). If the association between observations on the same cluster is not accounted for in the analytic strategy, inference associated with the estimated parameters may be erroneous [17,18].

We define $Y_{ij}$ as the binary response ($Y_{ij}=1,$ COVID-19 positive or $Y_{ij}=0$ otherwise) for the $j^{th}$ HCW in $i^{th}$ hospital. It is natural to model binary response as a binomial regression with a logit link. The marginal probability of an HCW testing positive for COVID-19 infection $\mu_{ij}=\mathrm{Pr}\left(Y_{ij}=1\right)$, is defined as:

$$\mathrm{logit}\left(\mu_{ij}\right)=x_{ij}^{\prime }\beta$$

where $x_{ij}$ represent the vector of risk factors (e.g., age, sex, type of HCW) and β is the vector of regression coefficients to be estimated [19,20].

The marginal log pair-wise odds ratio (POR) between two pairs of responses from two different hospitals ($j\neq j^{\prime }$) is modeled as:

$$\log\mathrm{POR}=\mathrm{logit}\;P\left(Y_{ij},Y_{ij^{\prime }}\right)=z_{ijj^{\prime }}^{\prime }\alpha$$

where $z_{ijj^{\prime }}^{\prime }$ = 1 if the pair of observations is from the same hospital ($j=j^{\prime }$) and 0 otherwise; α is the vector of association parameters to be estimated.

The ALR parameters, β and α, in the marginal response model and the pair-wise association were estimated simultaneously via solutions to the estimating equations:

$$S_{1}\left(\beta ,\alpha \right)=\overset{m}{\underset{i=1}{\sum }}\frac{\partial \mu_{i}^{\prime }}{\partial \beta }V_{i}^{-1}\left(Y_{i}-\mu_{i}\left(\beta \right)\right)=0\;S_{2}\left(\beta ,\alpha \right)=\overset{m}{\underset{i=1}{\sum }}\frac{\partial \xi_{i}^{\prime }}{\partial \alpha }W_{i}^{-1}\left(Y_{ijj^{\prime }}-\xi_{ijj^{\prime }}\right)=0$$

$$S_{2}\left(\beta ,\alpha \right)=\overset{m}{\underset{i=1}{\sum }}\frac{\partial \xi_{i}^{\prime }}{\partial \alpha }W_{i}^{-1}\left(Y_{ijj^{\prime }}-\xi_{ijj^{\prime }}\right)=0$$

where $\xi_{i}$ is a vector with elements $\xi_{ijj^{\prime }}=P\left(Y_{ij}=1|Y_{ij^{\prime }}=y_{ij^{\prime }}\right)$; $V_{i}$ is the variance-covariance matrix of $Y_{i}$ and $W_{i}$ is the diagonal matrix with elements $\xi_{ijj^{\prime }}\left(1-\xi_{ijj^{\prime }}\right)$ [17,18].

We fitted a univariable model and included all variables in the multivariate model. The results were presented as odds ratios (OR) for univariable models, adjusted odds ratios (aOR) for multivariable regression models, together with their 95% confidence intervals (CIs) and the POR for the within-cluster association. Multi-collinearity check was performed for the multivariable models, and no issue was found.

All data analyses were conducted in SAS 9.4 (SAS Institute Inc., Cary, NC, USA) and models implemented in SAS procedures PROC GENMOD.

## 3. Results

### 3.1. Baseline Characteristics

A total of 318 HCWs from six major healthcare facilities in Adamawa state, Nigeria, participated in this study. Table 1 presents the summary characteristics of the study sample at baseline. Of the 318 HCWs, 53 (16.67%) reported testing positive for COVID-19. The majority of HCWs were clinical staff, 237 (74.76%), and the remaining 80 (35.24%) were non-clinical staff. The proportion of clinical staff infected with COVID-19 was significantly higher than that of non-clinical staff (47 (19.83%) vs. 6 (7.50%), *p* = 0.011). With an overall mean (SD) age of 36.81 (8.98) years, HCWs who tested positive for COVID-19 were slightly older (*p* = 0.0081). Although the study sample consisted of slightly more males, 178 (55.97%), than females, 140 (44.03%), there was no significant gender difference between those who tested positive or negative. A higher proportion of the HCWs were married, 199 (62.58%), or had tertiary education, 263 (82.70%).

Table 2 describes the exposure risk and COVID-19 infection among HCWs. About one-third of the HWCs have 11–20 contacts with other people daily or spend up to 10 min attending to patients. More than half of the HCWs reported not being trained in infectious disease control in the last six months before the study, 184 (57.86%) or not having cared for a suspected COVID-19 patient, 205 (64.47%), or jobs did not involve handling aerosol producing procedures, 185 (58.18%). A significantly higher number of COVID-19 positive HCWs, 11 (37.93%) spent more than 40 min attending to patients than at other time intervals; cared (vs. not) for suspected COVID-19 patients (25% vs. 12.20%); reported not having adequate PPE (20.77% vs. 9.09%); reuse PPE (50.56% vs. 8.74%); not wearing N95 marks (21.54% vs. 9.48%); not using gloves (41.46% vs. 13.75%); not practicing hand hygiene consistently (31.82% vs. 14.29) and had recently travelled (35.29% vs. 15.67). 

### 3.2. Risk Factor for COVID-19 among HCWs 

**Figure 2:**
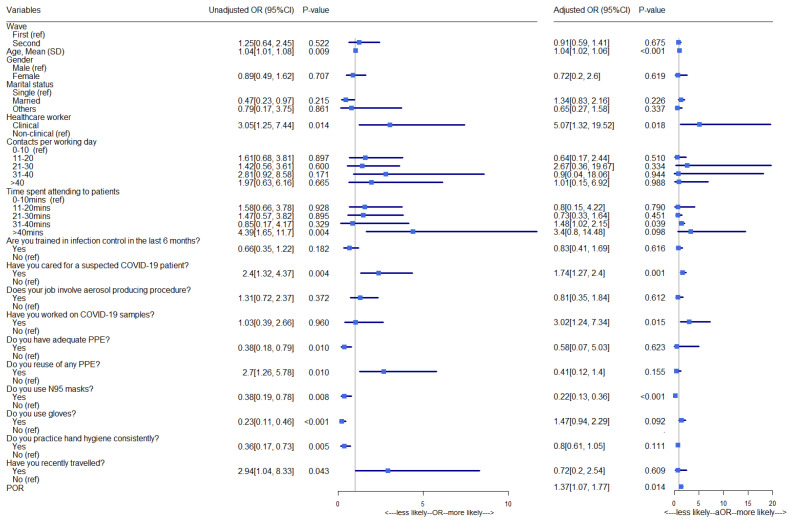
Association (univariable and multivariable) between COVID-19 infection and HCWs baseline characteristics and risk of exposure.

Results from unadjusted and adjusted models are presented in Figure 2. The models accounted for clustering at the hospital level via pair-wise odds ratio (POR). The univariable model revealed a significantly higher risk of COVID-19 infection (OR = 3.05, 95%: 1.25–7.44) among clinical staff compared with non-clinical staff. The was an increased risk of COVID-19 infection for age (OR = 1.04, 95%: 1.01–1.08). The odds for COVID-19 infection were four times higher (OR = 4.39, 95% CI: 1.65–11.70) for HCWs spending more than 40 min with patients than those who spent about 10 min. Significant associations for testing positive for COVID-19 were observed for caring for a suspected COVID-19 patient, no adequate PPE; reuse of any PPE; not using N95 masks; not using gloves; not practicing hand hygiene consistently, and recent travel. Specifically, having cared for suspected COVID-19 patients more than doubled the odds of being infected (OR = 2.40, 95% CI: 1.32–4.37), HCWs who reported having adequate PPE are less likely to test positive for COVID-19 (OR = 0.38, 95% CI: 0.18–0.79), while those who reported reusing their PPE are almost three times likely to get COVID-19 (OR = 2.70, 95% CI: 1.26–5.78) compared to those who do not reuse their PPE. Similarly, the following actions significantly reduce the chance of HCWs contracting COVID-19, use of N95 masks (OR = 0.38, 95% CI: 0.19–0.78) and gloves (OR = 0.23, 95% CI: 0.11–0.46)), and consistent practice of hand hygiene (OR = 0.36, 95% CI: 0.17–0.73). On the other hand, HCWs who reported recently travelling outside their city are about three times more likely to get COVID-19 (OR = 2.94, 95% CI: 1.04–8.33).

With regard to the adjusted model, age, time spent attending to patients, having cared for suspected COVID-19 patients, having worked on COVID-19 samples, and type of HCW were significantly associated with COVID-19 infection. While the effect size of age remained unchanged, the risk of COVID-19 infection has increased five-fold (aOR = 5.07, 95% CI: 1.32–19.52) for clinical staff compared to non-clinical staff after adjustment for baseline characteristics and exposure variable. Additionally, the model indicated a significant association between pairs of observations from HCWs within the same hospital (POR = 1.37, 95% CI: 1.07–1.77).

## 4. Discussion

This study examined the risk factors associated with COVID-19 infection among 318 HCWs in North-Eastern Nigeria. The overall prevalence of COVID-19 was 16.67% among HCWs. This estimate is slightly higher than the 15.2% reported among HCWs in the South-South region as of February 2021 [9]. Other studies have reported a similar prevalence of COVID-19 infection among HCWs; 16.32% in India [21], 20% in Italy [22], and 15% among Spanish HCWs [23]. In contrast, Al-Abri et al. [24] reported an incidence rate of 1.47% as of January 2021 among 847 HCWs in Oman and 6.03% in Australia [25]. Similar to previous studies [17,18], infection rates were observed to vary significantly between clinical and non-clinical staff (19.83% vs. 7.5%). Our results support the hypothesis that clinical staff are at a higher risk of infection compared to non-clinical HCWs. The degree of exposure is the scientific rationale underpinning this phenomenon. Non-clinical HCWs usually have little or no healthcare interaction with the patient. Several studies corroborate this plausible assertion [22,26,27].

Our univariate analyses suggest a significant association between COVID-19 infection among HCWs and the following factors: age, time spent attending to patients, caring for COVID-19 patients, adequacy of PPE, reuse of PPE, use of N95 masks, use of gloves, hand hygiene and recent travel.

As the COVID-19 pandemic ravages with an unprecedented surge in new infections and mortalities globally, the demand for PPEs has outpaced the available supplies. The shortage of PPEs caused an increase in occupational hazards for HCWs [8]. Production and supply chains were disrupted [3] as the global economy gradually shut down, and consequently, a shortage of PPEs ensued. The inadequate supply of PPEs led to their being reused. Similarly, the recycling of PPEs presented another risk of infection. Recycling, which has become almost a universal practice, is associated with the risk of self-contamination and nosocomial transmission [8]. The reuse of PPEs has been shown to pose more risk than inadequate availability of PPEs [28]. Recycling PPE poses the risk of self-contamination, especially during donning and doffing [29,30]. In addition, the integrity and safety of PPE is compromised due to the natural wear and tear phenomenon with extended usage beyond manufacturers’ recommendation [8]. The availability of gloves is a very basic shield in preventing nosocomial infections.

Time spent attending to COVID-19 patients is yet another risk factor identified in our study. In this study, prolonged contact time of more than 40 min between the patient and the HCW increases the risk of COVID-19 infection. This is consistent with Baker et al. [31], which reported that a prolonged contact time of more than two hours increases the risk of an HCW becoming infected. However, in their study, less than five per cent of the HCWs who had an average contact time of about 45 min and were initially unmasked eventually became positive [31].

In the multivariable analysis, being clinical staff was associated with a significant increase in the risk of COVID-19 among HCWs. We found clinical HCWs to be at a 5-fold increased risk of contracting COVID-19 than non-clinical HCWs. Nguyen et al. [8] found that frontline HCWs in the COVID-19 pandemic face a 12-fold increased risk compared to the general community in a combined UK and USA study, while Quigley et al. [25] estimated that HWCs are 2.69 times more likely to contract COVID-19 than the general community in Australia [25]. Consistent with our study, an Italian study showed a higher risk of COVID-19 in clinical HCWs than in non-clinical HCWs such as technicians [22]. In contrast, an Indian [21] and Spanish [23] study found no infection risk between non-clinical and clinical HCWs. Even though our study participants were predominantly young and middle-aged, it also brought this phenomenon to bear in that it has shown a significant relationship between increasing age and COVID-19 susceptibility. Biologically, as age increases, immunity depreciates. With the above finding, the principles of ergonomics should be applied by occupational health policy makers when assigning roles in healthcare settings in the face of infectious epidemics.

The major strength of this study is being a multicenter study involving both public and private health facilities. It derives uniqueness by being the first and the only study to date to have evaluated the risk profile of COVID-19 among HCWs in the North-Eastern region of Nigeria. The use of alternating logistic regression is another advantage and uniqueness of this study. We were able to simultaneously estimate the regression parameters and model the pair-wise association of the outcome variable. The significance of hospital-level clustering suggested that HCWs in the same hospital exhibited the same likelihood of testing positive for COVID-19. It provides the healthcare policymakers with empirical evidence in planning for infection, prevention and control (IPAC).

This study is not without limitations. Firstly, some HCWs who tested positive declined consent for fear of stigmatization. Secondly, the study may be prone to the inherent recall bias that is associated with a retrospective study. Thirdly, while this study sought to explore the usage of PPEs, it stopped short of scrutinizing whether the HCWs adhered strictly to the standard procedure and guidelines guiding the usage of the PPEs or not. Furthermore, it also fell short of integrating quality control checks in assessing PPEs nor examined the consistency in the use of PPEs by HCWs.

## 5. Conclusions

In summary, this study presents the disparities in the prevalence of COVID-19 between clinical and non-clinical HCWs in North-East Nigeria. We showed that COVID-19 infection is higher among clinical HCWs than in non-clinical HCWS. We also showed that the reuse or recycling of PPEs, which became an almost universal practice during the explosive first wave, carries a significant risk (in the unadjusted model) for the HCWs.

In this regard, holistic pandemic management requires an occupational risk assessment as part of the mitigation strategy to inform containment policies and risk management protocol. We recommend that health policymakers make policies that will guarantee an adequate supply of PPEs, especially the N95 mask for the clinical HCWs. HCWs who arrive from high endemic areas of the COVID-19 disease should isolate for about one week before resuming work to avoid cross-infection and asymptomatic transmission. We also advocate for a full complement of risk assessment, stratification, and communication to be institutionalized universally in the healthcare facilities in the subsequent health policy framework.

Since this study has shown that prolonged contact time is a potential risk of infection, it is recommended that capacity building in terms of staffing and telehealth capabilities to reduce contact time is necessary. Similarly, intensive public education and reorientation with the view to correcting the misperception and stigmatization of COVID-19 disease and vaccination should be enhanced. Finally, we advocate for the strengthening surveillance infrastructure and the establishment of epidemic intelligence units. Future research could focus on a systematic serological survey and large-scale genome sequencing of COVID-19 among HCWs, and society as a whole, as this will give the true burden and trail the spread of this pandemic.

## Figures and Tables

**Table 1:** Description of HCWs’ baseline characteristics and COVID-19 infection status.

Variable	N	%	COVID-19		*p*-Value ^†^
			Positive	Negative	
Overall	318	100.00	53 (16.67)	264 (83.02)	
Waves					
First	244	76.97	39 (15.98)	205 (84.02)	0.521
Second	73	23.03	14 (19.18)	59 (80.82)	
Healthcare worker					
Clinical	237	74.76	47 (19.83)	190 (80.17)	**0.011**
Non-clinical	80	25.24	6 (7.50)	74 (92.50)	
Age (Years) Mean ± SD	36.81 ± 8.98		39.83 (9.57)	36.22 (8.87)	**0.008**
Gender					
Male	178	55.97	31(17.42)	147 (82.58)	0.707
Female	140	44.03	22 (15.83)	117 (84.17)	
Marital status					
Single	103	32.39	11 (10.68)	92 (89.32)	
Married	199	62.58	40 (20.20)	158 (79.80)	0.099
Others	16	5.03	2 (12.50)	14 (87.50)	
Education					
Primary	4	1.26	0 (0.00)	4 (100)	
Secondary	51	16.04	6 (11.76)	45 (88.24)	0.371
Tertiary	263	82.70	47 (17.94)	215 (82.06)	

**^†^** Based on chi-square test; Bold *p* < 0.05.

**Table 2:** Exposure risk and COVID-19 infection status among HCWs.

Variable	N	%	COVID-19		*p*-Value ^†^
			Positive	Negative	
Contacts per working day					
0–10	74	24.75	9 (12.16)	65 (87.84)	0.438
11–20	99	33.11	18 (18.18)	81 (81.82)	
21–30	73	24.41	12 (16.44)	61 (83.56)	
31–40	25	8.36	7 (28.00)	18 (72.00)	
>40	28	9.36	6 (21.43)	22 (78.57)	
Time spent attending to patients					
0–10 min	90	34.09	11 (12.22)	79 (87.78)	**0.029**
11–20 min	73	27.65	13 (18.06)	50 (81.94)	
21–30 min	53	20.08	9 (16.98)	44 (83.02)	
31–40 min	19	7.2	2 (10.53)	17 (89.47)	
>40 min	29	10.98	11 (37.93)	18 (62.07)	
Are you trained in infectious disease control in the last 6 months?					
Yes	134	42.14	18 (13.43)	116 (86.57)	0.18
No	184	57.86	35 (19.13)	148 (80.87)	
Have you cared for a suspected COVID-19 patient?					
Yes	113	35.53	28 (25.0)	84 (75.0)	**0.003**
No	205	64.47	25 (12.20)	180 (87.80	
Does your job involve aerosol producing procedure?					
Yes	133	41.82	25 (18.94)	107 (81.06)	0.371
No	185	58.18	28 (15.14)	157 (84.86)	
Have you worked on COVID-19 samples?					
Yes	42	17.87	6 (14.29)	36 (85.96)	0.96
No	193	82.13	27 (13.99)	166 (86.01)	
Do you have adequate PPE?					
Yes	110	34.59	10 (9.09)	100 (90.91)	**0.008**
No	208	65.41	43 (20.77)	164 (79.23)	
Do you reuse any PPE?					
Yes	214	67.3	44 (20.56)	170 (79.44)	**0.008**
No	104	32.7	9 (8.74)	94 (91.26)	
Do you use N95 masks?					
Yes	116	37.3	11 (9.48)	105 (90.52)	**0.006**
No	195	62.7	42 (21.54)	153 (78.76)	
Do you use gloves?					
Yes	241	85.46	33 (13.75)	207 (86.25)	**<0.001**
No	41	14.54	17 (41.46)	24 (58.54)	
Do you practice hand hygiene consistently?					
Yes	274	86.16	39 (14.29)	234 (85.71)	**0.004**
No	44	13.84	14 (31.82)	30 (68.18)	
Have you recently travelled?					
Yes	17	5.35	6 (35.29)	11 (64.71)	**0.035**
No	301	94.65	47 (15.67)	253 (84.33)	

**^†^** Based on the chi-square test: Bold *p* < 0.05.

## Data Availability

The data for this study are available from the first author upon reasonable request.

## Supplementary Materials

The following supporting information can be downloaded at: https://www.mdpi.com/article/10.3390/healthcare10101919/s1, Table S1: Questionnaire.
