# Supplementary material for: Risk Factors for COVID-19 Infection among Healthcare Workers in North-East Nigeria
Source: Healthcare (Basel). 2022 Sep 30;10(10):1919. doi: 10.3390/healthcare10101919 (PMC9601610; doi:10.3390/healthcare10101919)
Supplement: Supplementary file 1 [file healthcare-10-01919-s001.zip › healthcare-1892201-supplementary.pdf]

Table S1: Questionnaire

EPIDEMIOLOGICAL PROFILE OF COVID-19 INFECTIONS AMONG HEALTHCARE WORKERS IN A NORTH EASTERN STATE OF NIGERIA

Please INDICATE **NA** (NOT APLLICABLE) IF A QUESTION DOES NOT APPLY TO YOU

HCW- healthcare worker

Hospital/health facility --------------------

Social demographics

1. **Age** (yrs.) ------------------
2. What is your sex?

   (i) Male [ ] (ii) Female [ ]
3. **What is your marital status?**

   (Single [ ] (ii) Married [ ] (iii) Divorced [ ] (iv) Widowed [ ] (v) Separated [ ]
4. **What is your ethnic group?**

(i) Fulani [ ] (ii) Hausa[ ] (iii)Igbo [ ] (iv)Yoruba [ ] (v)Marghi { } (vi)Michika{ } (vii) Bachama/ Mbula/Bwate{ } (viii) Kilba { } (ix) Lunguda { } (x) Chamba{ }

(viii) Others, please specify--------------

5. **What is your highest level of education?**

(i)Primary school [ ] (ii) Secondary school [ ] (iii) Tertiary [ ] (iv) Others, please specify--------------

6. **Professional category**

(i)Medical doctor { } (ii) Nurse/midwife { } (iii)Medical Lab scientist { }

(iv)Pharmacist { } (v) cleaner/ward attendant{ } (vi) administrator/Accountant{ }

(vii) CHEW/CHW{ } (viii)Mortician { } (ix)lab technician{ }

(x)Radiographer/Radiology technician { } (xi) biomedical engineer/ technician { }

(xii) Medical record officer { } (xiii) security officer { } (xiv) Optometrist { }

(xv) ECG Technician { }

7. **Department/ primary area of assignment/ work station**

(i)Accident and emergency { } (ii)nursing{ } (iii)laboratory{ } (iv)Pharmacy{ } (v)Anesthesia{ } (vi)medicine { } (vii)surgery{ } (viii)Pediatrics { } (ix) Obs& Gyn { } (x) Histopathology /Morgue { } (xi) GOPD { } (xii) PHC{ }

(xiii) COVID-19 treatment/Isolation center { } (xiv) finance/admin { }

(xv) Labor ward/ antenatal/post-natal ward { } (xvi) Radiology { } (xvii) Theater/ ICU { } (xviii) Dialysis center { } (xix) Laundry { } (xx) public health { }

(xxi) PCR/biosafety lab { } (xxii) medical record { } (xxiii) security post

(xxiv) Opthalmology { } (xxv) ECG room

8. **Do you work in the private health sector** (i) Yes { } (i) No { }

9. **Designation/Rank --**

10. **For how long have been working as a healthcare worker (**i) 0 -3yrs { }

(ii) 4– 7yrs{ } (iii) 8- 11yrs { } (iv) 12- 15yrs { } (v) >15 yrs

11. Category of employment (i) temporary { } (ii) contract { } (iii) permanent staff{ }

12. **When did you the take the covid-19 test (i) April { } (ii) May { } (iii) June** { } (iv) July { } (v) August { } (vi) Sept { } (vii) Octo { } (viii) Nov { }

(ix) Dec { } (x) Jan 2021 { }

13.What was the outcome of your test? (i) Positive { } (ii) Negative { }

(iii) Inconclusive { }

14. Why did you go for the COVID-19 test? (i) exposure to a confirmed case { } (i)exposure to suspicious case{ } (iii) contact with a confirmed HCW{ } (iv) for health promotion purpose{ } (v) following symptoms of covid-19 (vi) managerial directive following a positive staff in a your unit{ }

15- **Did you have an immediate family member who had been diagnosed with covid-19 around the time of your diagnosis** (i) yes { } (i) No { }

16. **Duration of your working hours {duty hour} per week ----------------------**

17**. Have you been trained in infection, prevention control in the last 6months? (i) Yes { }** how many times? ----------------------- **(ii) No { } --**

18. Which of these **personal protective equipment PPE were you using consistently before your covid-19 test? (**i) Surgical mask { } (ii) N 95 { } (iii) cloth/fabric mask { } (iv) hand sanitizer { } (v) Hand gloves { } (vi) None

19. Did you always use hand gloves while attending to patients or in between patients prior to your covid-19 test? –(i) yes { } (ii) No { }

20. **Did you practice hand hygiene consistently about the time of covid-19 test? (i) yes { } (ii) No { } ---------------------**

21. **Did you engage in a recent travel at about the time you were tested for covid-19?**

(i) yes { } (ii) No { }

22**. Which of these Symptoms did you experience?**

(i) cough { } (i) fever{ } (iii)difficulty in breathing{ } (iv)sore throat{ } (v) loss of smell{ } (vi) loss of taste{ } (vii) generalized body pains{ } (ix) diarrhea { } (x)Vomiting { } (xi)catarrh and nasal congestion { } (xii) others{ } (xiii) none { }

23. **Does your job routine involves aerosol producing procedures (APP) such as nebulization, airway suctioning, CPR, endotracheal intubation, high flow oxygen administration? (i) Yes { } (ii) No { }**

24. Do you have any of these comorbidities or conditions (i) diabetes { } (ii) hypertension{ (iii) asthma{ } (iv)obesity { } (v) prolonged steroid use{ } (vi) cancer { } (vi) none{ }

25. Did you care for a confirmed or suspected covid-19 patient prior to your covid-19 test (i)Yes { } (ii) No { } **or worked on a biological sample of a confirmed covid-19 patient ?** (i) Yes { } (ii) No { }

26- **Did you have the adequate personal protective equipment PPE which you needed to work with while working at that time?** (i) Yes { } (ii) No { }

27. **Did you have to reuse any personal protective equipment PPE?** Such as gloves, face mask, gown, face shield? (i) Yes { } (ii) No { }

28. **Did you require admission or you self-isolated following your covd-19 positive rest? [Underline which applies to you]**

29. **What was your duration of hospital admission? ---------------**

30. While on admission did you require (i) oxygen { } (ii) Ventilator { } (iii) None { }

31. What do you think was your risk factor for contracting covid-19?

(i) Lack of enough PPE { } (ii) unawareness of infection, prevention and control methods { } (iii) overly long working hours { } (iv) Poor safety culture on your part { }

32. Average number of close contacts per working day (i) 0-10 persons { } (ii) 11- 20 persons { } (iii) 21 -30 { } (iv)31-40 { } (v)41-50 { } (vi) 51 and above

33. What is the average duration of time you spend when attending to a patient or analyzing covid-19 biological sample [applies to Lab scientist working in the biosafety lab only]

(i) 0- 10mins { } (ii)11-20mins { } (iii)21-30mins { } (iv)31-40mins { } (vi) 41mins and above
